# An open-label study to determine the maximum tolerated dose of the multitargeted tyrosine kinase inhibitor CEP-11981 in patients with advanced cancer

**DOI:** 10.1007/s10637-014-0147-9

**Published:** 2014-08-26

**Authors:** Roberto Pili, Michael Carducci, Peter Brown, Herbert Hurwitz

**Affiliations:** 1 Roswell Park Cancer Institute, Elm and Carlton Streets, Buffalo, NY 14263 USA; 2 Johns Hopkins Sidney Kimmel Comprehensive Cancer Center, Baltimore, MD USA; 3 Teva Branded Pharmaceutical Products R&D, Inc, Frazer, PA USA; 4 Duke Cancer Institute, Duke University, Durham, NC USA

**Keywords:** Dose-finding study, Multitargeted inhibition, Safety profile, Tie-2, Tyrosine kinase inhibitor, Vascular endothelial growth factor

## Abstract

*Background* This phase I study evaluated the pharmacokinetics and pharmacodynamics of CEP-11981, an oral vascular endothelial growth factor (VEGF) tyrosine kinase inhibitor, in patients with advanced, relapsed, or refractory solid tumors. *Methods* Oral CEP-11981 dose escalations followed a modified Fibonacci sequence (from 3.0 to 4.2, 5.9, 11.8, 19.7, 29.6, 41.4, 55.0, 73.0, 97.4, and 126.6 mg/m^2^). The maximum-tolerated dose (MTD), dose-limiting toxicities (DLTs), tumor response, and safety were evaluated. *Results* CEP-11981 was tolerated at doses between 3.0 and 97.4 mg/m^2^. The MTD of CEP-11981 was determined to be 97.4 mg/m^2^, with DLTs observed at the 126.6 mg/m^2^ dose. The DLTs were grade 4 neutropenia in 1 patient and grade 3 T-wave inversion with chest heaviness and fatigue in 1 patient. All 3 events resolved on stopping CEP-11981. The most frequently reported adverse events of any grade were fatigue, nausea, diarrhea, decreased appetite, abdominal pain, back pain, vomiting, constipation, headache, dizziness, and dyspnea. Treatment-related grade 3/4 neutropenia was observed in the highest-dose cohorts (2 patients at 97.4 mg/m^2^ and 1 patient at 126.6 mg/m^2^), indicating some off-target inhibition. VEGF inhibition was greatest in the higher-dose groups. Although no patient experienced complete or partial response, 44 % patients achieved stable disease when measured at ≥ 6 weeks, which occurred more frequently in cohorts receiving ≥ 73.0 mg/m^2^. *Conclusions* In patients with recurrent or refractory solid tumors, disease stabilization was achieved. Despite acceptable tolerability of CEP-11981 at the MTD, further development by the sponsor has ceased.

## Introduction

Angiogenesis plays an essential role in the development and progression of cancer [1]. Numerous proangiogenic signaling cascades have been identified, such as the vascular endothelial growth factor (VEGF) ligands and their respective receptors (R), VEGFR-1/Flt-1, VEGFR-2/KDR, and VEGFR-3/Flt-4 [2–4]. Notably, the VEGFR-2/KDR subtype in particular plays a primary role in promoting angiogenesis.

Bevacizumab, a humanized monoclonal anti-VEGF A antibody, abrogates signal transduction of the proangiogenic VEGFR-1 and VEGFR-2 [5]. Bevacizumab, alone and in combination, has demonstrated the ability to block or attenuate tumor growth and in some tumor types, improve overall survival (OS) and/or progression-free survival (PFS) [6–10]. However, the magnitude and duration of benefit has generally been modest because of numerous mechanisms of intrinsic and/or acquired resistance to antiangiogenic therapy [11–14]. Because tumor cells engage a wide range of angiogenic factors, agents that target a single factor or ligand-receptor axis may be insufficient [14]. With the objective of improving clinical outcomes and providing an oral medication for advanced cancer, antiangiogenic therapies that inhibit multiple signaling pathways, including other proangiogenic targets, were developed [13]. These include tyrosine kinase inhibitors (TKIs) that target the VEGF signaling pathway. However, the first approved VEGFR-TKIs (eg, sunitinib, sorafenib) lack specificity, and it has been postulated that the abrogation of the other signaling pathways would promote adverse events not associated with the main angiogenic signaling pathways. Therefore, other VEGFR-TKIs with improved potency and specificity for additional targets including proangiogenic platelet-derived growth factor (PDGF) and its receptors, PDGFR-α and PDGFR-β have potential clinical advantages [15, 16].

A proangiogenic signaling pathway and potential therapeutic target is the Tie-2 receptor and its ligands, angiopoietin (Ang)-1 and Ang-2 [17–19]. Studies suggest that the VEGFR and Tie-2 pathways are synergistic and promote a greater degree of angiogenesis versus either pathway alone [11, 20–22]. Therefore, it has been proposed that for the optimal inhibition of tumorigenesis, both VEGFR and Tie-2 should be simultaneously inhibited [21, 23]. Solid tumor cell-line experiments demonstrated that Tie-2 upregulates Ang-2 [19] and animal models have shown that relapsing tumors upregulate Ang-1 [11]. In a human melanoma xenograft model, inhibition of both the VEGFR-2 and Tie-2 pathways versus VEGFR-2 alone reduced the amount of angiogenesis and the tumor burden [23]. The potential value of a multitargeted inhibitor was recently borne out in a phase III study of regorafenib monotherapy in patients with treatment refractory metastatic colorectal cancer that showed modestly improved OS (6.4 versus 5.0 months; *P* = 0.0052) [24].

CEP-11981 is an orally active multitargeted VEGFR-TKI that inhibits VEGFR-1, VEGFR-2, Tie-2, fibroblast growth factor receptor-1, proto-oncogene c-SRC, and Aurora A (half maximal inhibitory concentration [IC_50_] of 3, 4, 22, 13, 37, and 42 nM, respectively) [25]. Preclinical studies have shown that CEP-11981 exhibits promising permeability, metabolic stability, and pharmacokinetic properties across multiple species [25]. Studies of pharmacologic activity across angiogenesis assays, animal tumor models, and human tumor models have shown sustained, dose-related antiangiogenic and antitumor inhibition [25]. This phase I study was conducted to determine the maximum-tolerated dose (MTD), dose-limiting toxicities (DLTs), pharmacokinetics, and pharmacodynamics of CEP-11981 in patients with advanced, relapsed/refractory solid tumors, with the objective to identify the recommended dose of CEP-11981 for use in a phase II study.

## Methods

### Patient selection

Adult patients (≥18 years) who had histologically or cytologically confirmed relapsed or refractory solid tumor that was unresponsive or poorly responsive to accepted treatment modalities, a life expectancy of at least 12 weeks, and an Eastern Cooperative Oncology Group (ECOG) performance score of 0–2 were included in this study. Eligible patients had a normal neurological examination and were fully recovered from any prior surgical procedures or had reversible side effects of prior cancer therapy. Patients were excluded if they had abnormal hematologic (absolute neutrophil count [ANC] < 1500/mm^3^, platelet count < 100,000/mm^3^, or hemoglobin <9 g/dL), hepatic (bilirubin > 1.5 times the upper limit of normal [ULN], alanine aminotransferase [ALT] or aspartate aminotransferase [AST] > 2.0 times the ULN in the absence of known hepatic metastases, or ALT or AST > 3.0 times the ULN in the presence of known hepatic metastases), or kidney functioning (creatinine value > 1.5 mg/dL). Other reasons for exclusion included cerebral metastases, known hypersensitivity to gelatin or lactose monohydrate, preexisting coagulopathy, recent hemoptysis, gross hematuria, gastrointestinal bleeding, history of a clinically significant cardiovascular or cerebrovascular event within 6 months of study entry, or blood pressure > 150 mmHg systolic or 90 mmHg diastolic with medication. Patients were excluded if they were currently receiving warfarin or heparin therapy; received any other antineoplastic treatment for solid tumors (hormonal treatment permitted) within the previous 4 weeks; received an investigational drug within the previous 4 weeks; or received a human cytochrome P450 (CYP) 1A2, CYP2C8, or CYP3A4 inducer within the previous 4 weeks.

Female patients who were pregnant or lactating were excluded from this study. All men capable of producing offspring and all women of childbearing potential were required to use reliable contraception.

### Study design and endpoints

This was an open-label, nonrandomized, multicenter, dose-escalation phase I study of CEP-11981 in patients with advanced, relapsed/refractory solid tumors. The study was conducted in accordance with Good Clinical Practice: Consolidated Guidance approved by the International Conference on Harmonisation, and applicable national and local laws and regulations and approved by appropriate Independent Ethics Committees or Institutional Review Boards. All patients provided written consent before study procedures or assessments were performed.

The primary measures were MTD and DLTs. MTD was defined as the highest dose at which one-third or fewer of patients in a cohort experienced a DLT, which included grade ≥ 2 proteinuria, grade ≥ 3 nonhematologic toxicity (excluding hypertension and pain), grade ≥ 3 thrombocytopenia with bleeding, or grade 4 hematologic toxicity that was not clearly due to progressive cancer. Secondary measures included the pharmacokinetics of CEP-11981 after a single dose and multiple doses, the pharmacokinetic-pharmacodynamic profile of CEP-11981, safety, and preliminary efficacy. The efficacy endpoint was the proportion of patients who achieved complete or partial response on study (minimum of 6 weeks) according to Response Evaluation Criteria in Solid Tumors (RECIST) guidelines [26].

Pharmacokinetic parameters calculated after a single dose of CEP-11981 included area under the plasma drug concentration versus time curve (AUC) from zero to infinity (AUC_0-∞_)_,_ to the last measurable concentration (AUC_0-t_), and to 24 h (AUC_0–24_); maximum observed plasma drug concentration (C_max_); time to maximum observed plasma drug concentration (T_max_); terminal elimination half-life (t_½_); and predicted accumulation ratio (R_pred_), which was defined as AUC_0–∞_ (day 1, cycle 1)/AUC_0–24_ (day 1, cycle 1). Pharmacokinetic parameters calculated after multiple doses included AUC_0-t_, C_max_, T_max_, and t_½_, and observed accumulation ratio (R_obs_). R_obs_ was defined as AUC for 1 dosing interval following multiple doses (AUC_τ_; day 15, cycle 1)/AUC_0–24_ (day 1, cycle 1). The pharmacokinetic analysis included patients who received ≥ 1 dose of CEP-11981 and had ≥1 pharmacokinetic value. Pharmacodynamics was assessed by VEGFR-2/KDR inhibitory activity in response to CEP-11981 treatment.

The safety of CEP-11981 with dose escalation was assessed by adverse events, clinical laboratory test results, vital signs, electrocardiogram (ECG), physical examination, and concomitant medication use. The safety analysis included all patients who received ≥ 1 dose of CEP-11981. Adverse events were recorded and graded according to the Common Terminology Criteria for Adverse Events, version 3.0.

### Patient evaluations

Physical examinations, urinalysis, ECG, and laboratory evaluations (including serum chemistry and hematology) were performed at screening and on days 1, 2, 8, 15, 22, and 29 of cycle 1; on days 1, 15, and 29 of subsequent cycles; and at the end-of-treatment follow-up visit (14 days after the last dose). Adverse events were evaluated on days 2, 8, 15, and 22 of cycle 1; on days 1, 15, and 29 of every subsequent cycle; and at the end-of-treatment follow-up visit. To evaluate preliminary efficacy, tumors were measured by computed tomography or contrast magnetic resonance imaging scans at screening and every 6 weeks.

### Study treatment and dose escalation

Oral CEP-11981 was administered once daily for the first 28 days of each 42-day cycle. Initial dose escalation from the starting dose of 3 mg/m^2^ was by 40 % increments. After completion of additional toxicology studies and the preliminary analysis of plasma exposure in patients, the dose for the fourth cohort was doubled from the third cohort and thereafter the escalation followed a modified Fibonacci sequence. The final dose levels were: 3.0, 4.2, 5.9, 11.8, 19.7, 29.6, 41.4, 55.0, 73.0, 97.4, and 126.6 mg/m^2^. The dose administered was rounded to the smallest capsule strength available. Dosing for a patient was to be stopped at the onset of a DLT and, at resolution within 14 days, could be resumed at a dose equal to 50 % of the patient’s current dose. In the event of DLTs in one-third or more of patients receiving 3.0 mg/m^2^, the dose would be reduced to 1.5 mg/m^2^. Dose escalation proceeded according to the standard 3 + 3 design.

### Pharmacokinetic studies

The venous blood samples for pharmacokinetic analysis were collected on days 1 and 15 of dosing during cycle 1 immediately before dose administration and at 0.5, 1, 1.5, 2, 3, 4, 6, 8, 12 (this sample was optional and could be collected from 10 to 14 h), and 24 h postdose, as well as on days 8, 22, 29, and 43. The venous blood samples for pharmacokinetic-pharmacodynamic analysis were collected on days 1 and 15 of dosing during cycle 1 immediately before dose administration and at 1 h postdose, and on day 43. At each draw, 2 samples were obtained in 6-mL sodium heparinized vacutainers. Plasma was obtained and stored at −70 °C until shipment to the sponsor for analysis.

### Pharmacodynamic bioassay

A bioassay with engineered chimeric porcine aortic endothelial (PAE) cell lines stably expressing a TrkA-VEGFR-2/KDR domain was used to evaluate human plasma-associated shifts in cellular IC_50_ for CEP-11981 inhibition of ligand-stimulated VEGFR-2/KDR phosphorylation. Plasma samples for the bioassay were obtained 1 h postdose and on days 1 and 15; they were collected from patients across cohorts.

## Results

### Patients

**Fig. 1:**
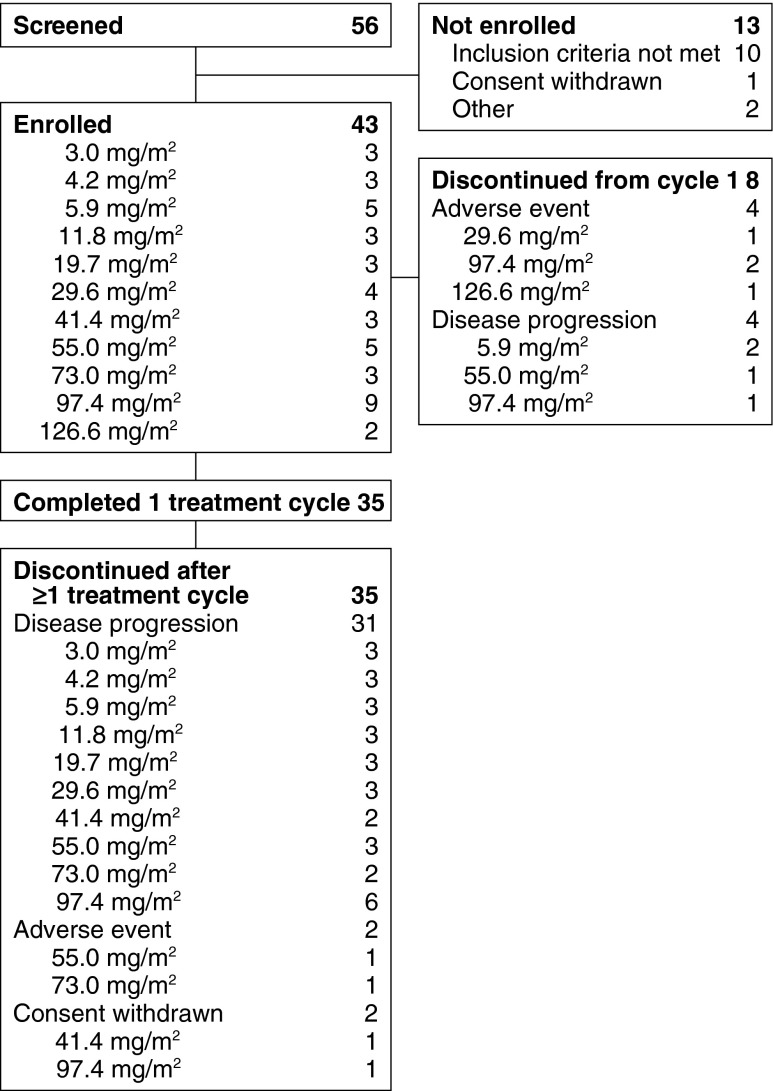
Patient Disposition. Shows that 56 patients were screened for the study and 43 patients enrolled. 10 patients did not meet inclusion criteria. Enrolled patients received CEP-11981 at dose levels ranging from 3.0 mg/m^2^ to 126.6 mg/m^2^; 8 patients discontinued treatment during cycle 1 due to adverse event (*n* = 4) or disease progression (*n* = 4). The remaining 35 patients discontinued after ≥1 treatment cycle due to disease progression (*n* = 31), adverse event (*n* = 2), or consent withdrawal (*n* = 2)

Forty-three patients with advanced, refractory/relapsed solid tumors were enrolled in the study (Fig. 1) between September 2007 and February 2011. Demographics and baseline characteristics are listed in Tables 1 and 2. Patients were heavily pretreated, with all patients having received prior radiotherapy (100 %), chemotherapy (100 %), and surgery (100 %). The most common cancers in this study population were colorectal (19 %) and lung (19 %). Thirty-five patients (81 %) completed 1 treatment cycle and 17 completed ≥ 2. During the first cycle, 8 patients discontinued due to adverse events (*n* = 4) and disease progression (*n* = 4). Seventeen patients (40 %) received ≥ 2 cycles. Among patients who received ≥ 1 cycle, 35 (81 %) discontinued, most commonly for disease progression (31 patients, 72 %).

**Table 1:** Patient demographics and baseline characteristics

Variable	Total (*N* = 43)
Median age, years (range)	63 (26–77)
Sex, *n* (%)	
Male	22 (51)
Female	21 (49)
ECOG, *n* (%)	
0	21 (49)
1	21 (49)
2	1 (2)
Median years since first cancer diagnosis (range)	3.2 (0.6–17.8)
Best response to prior cancer therapy, *n* (%)	
Complete	3 (7)
Partial	7 (16)
Stable disease	23 (53)
Disease progression	7 (16)
Missing	3 (7)
Most common tumor type, *n* (%)	
Colorectal	8 (19)
Lung	8 (19)
Prior radiation therapy	43 (100)
Prior chemotherapy^a^	43 (100)
Most common chemotherapies, *n* (%)	
Bevacizumab	18 (42)
Gemcitabine	16 (37)
Cisplatin	13 (30)
5-Fluorouracil, irinotecan, oxaliplatin	11 (26)
Carboplatin, docetaxel	10 (23)
Capecitabine, leucovorin	9 (21)
Cetuximab	8 (19)
Doxorubicin	7 (16)
Paclitaxel	6 (14)
Range of cycles per regimen	2–29
Prior surgery	43 (100)

^a^Names as recorded in listing of prior therapies

*ECOG*, Eastern Cooperative Oncology Group

**Table 2:** Tumor type by cohort

3.0 mg/m^2^ *n* = 3	4.2 mg/m^2^ *n* = 3	5.9 mg/m^2^ *n* = 5	11.8 mg/m^2^ *n* = 3	19.7 mg/m^2^ *n* = 3	29.6 g/m^2^ *n* = 4	41.4 mg/m^2^ *n* = 3	55.0 mg/m^2^ *n* = 5	73.0 mg/m^2^ *n* = 3	97.4 mg/m^2^ *n* = 9	126.6 mg/m^2^ *n* = 2
• Endometrial • GIST • TCC of bladder	• Primitive neuroectodermal • Osteogenic sarcoma • Head	• Ovarian • Primary serous adenocarcinoma • Breast • Duodenal • Renal	• Sarcoma • Adrenal • Colorectal	• Pancreatic • Colorectal • Head	• Colorectal (2) • Head • Renal	• Chondrosarcoma • Breast • Colorectal	• Lung (3) • Chondrosarcoma • Prostate	• Pancreatic • Lung • Prostate	• Lung (4) • Colorectal • Ocular • Unknown primary • Head • Renal	• Colorectal (2)

*GIST*, gastrointestinal stromal tumor; *TCC*, transitional cell carcinoma

### Dose escalation and MTD

DLTs did not occur at lower doses and occurred only in the 126.6 mg/m^2^ dose cohort. The first 2 patients in the cohort had DLTs and recruitment at this dose level was stopped. One patient experienced grade 4 neutropenia and the second patient experienced grade 2 exertional dyspnea, grade 2 chest heaviness, and grade 3 new T-wave inversion. The second patient with colorectal cancer (and without a history of active cardiovascular disease), was hospitalized for cardiac work-up with probable ischemia, and the ECG changes and chest discomfort resolved. Three additional patients were added to the 97.4 mg/m^2^ cohort. As no DLTs were observed in the 97.4 mg/m^2^ cohort, this dose was determined to be the MTD.

### Exposure

All patients who received ≥ 1 dose of study drug (*n* = 43) were evaluated for safety. These patients received between 1 and 10 treatment cycles of CEP-11981, with a median of 28 days (range, 5–250) of treatment (Table 3). The overall median relative dose intensity across all cycles was 96.8 % (range, 1.6 % to 107.5 %). Four patients received ≥ 5 cycles: 1 in the 3.0 mg group (8 cycles, endometrial cancer), 1 in the 29.6 mg group (6 cycles, head), 1 in the 55.0 mg group (10 cycles, lung), and 1 in the 97.4 mg group (5 cycles, lung).

**Table 3:** Study drug exposure by cohort

	3.0 mg/m^2^	4.2 mg/m^2^	5.9 mg/m^2^	11.8 mg/m^2^	19.7 mg/m^2^	29.6 mg/m^2^	41.4 mg/m^2^	55.0 mg/m^2^	73.0 mg/m^2^	97.4 mg/m^2^	126.6 mg/m^2^	Total
Median days treated (range)	*n* = 3	*n* = 3	*n* = 5	*n* = 3	*n* = 3	*n* = 4	*n* = 3	*n* = 4	*n* = 3	*n* = 9	*n* = 2	*N* = 42
	84.0 (28.0–220.0)	28.0 (27.0–56.0)	28.0 (24.0–55.0)	28.0 (27.0–56.0)	56.0 (28.0–84.0)	46.0 (17.0–148.0)	28.0 (28.0–84.0)	70.0 (27.0–250.0)	28.0 (28.0–93.0)	28.0 (5.0–140.0)	14.5 (7.0–22.0)	28.0 (5.0–250.0)
Mean (SD) number of cycles	*n* = 3	*n* = 3	*n* = 5	*n* = 3	*n* = 3	*n* = 4	*n* = 3	*n* = 5	*n* = 3	*n* = 9	*n* = 2	*N* = 43
	4.0 (3.61)	1.3 (0.58)	1.2 (0.45)	1.3 (0.58)	2.0 (1.00)	2.8 (2.36)	1.7 (1.15)	3.4 (3.91)	2.0 (1.73)	1.9 (1.36)	4.0 (4.24)	2.2 (2.10)
Mean (SD) total dose, mg	*n* = 3	*n* = 3	*n* = 5	*n* = 3	*n* = 3	*n* = 4	*n* = 3	*n* = 4	*n* = 3	*n* = 9	*n* = 2	*N* = 42
	332.0 (296.22)	155.4 (69.14)	187.6 (77.42)	436.6 (194.25)	1,103.2 (551.60)	1,901.8 (1,750.39)	1,932.0 (1,338.53)	5,733.8 (5,775.85)	3,625.7 (2,739.53)	4,599.4 (4,163.72)	2,372.6 (583.50)	2,389.9 (3,242.13)
Mean (SD) RDI, %	*n* = 3	*n* = 2	*n* = 5	*n* = 3	*n* = 3	*n* = 4	*n* = 3	*n* = 4	*n* = 3	*n* = 9	*n* = 2	*N* = 41
	96.2 (2.27)	90.7 (1.78)	94.1 (9.27)	98.0 (4.27)	95.9 (3.42)	81.8 (18.60)	99.1 (2.30)	94.0 (19.01)	99.8 (7.24)	75.0 (32.75)	38.6 (52.30)	87.2 (23.62)

*RDI*, relative dose intensity; *SD*, standard deviation

### Adverse events

All 43 patients experienced ≥ 1 adverse event, and 38 patients (88.3 %) were deemed to have had adverse events possibly, probably, or definitely related to study drug. The most frequently reported adverse event of any grade was fatigue (*n* = 22, or 51 %). Other frequently reported adverse events (≥20 % of patients) were nausea (47 %), diarrhea (33 %), decreased appetite (33 %), abdominal pain (30 %), back pain (28 %), vomiting (28 %), constipation (28 %), headache (28 %), dizziness (28 %), and dyspnea (23 %). These adverse events were reported at a similar frequency between dosage cohorts, and no relationship with dose was evident. Most adverse events were grade 1 or 2.

Grade 3 or 4 adverse events occurred in 16 (37 %) patients across dosing cohorts (14 [32.6 %] grade 3 and 2 [4.7 %] grade 4). Treatment-related grade 3 or 4 adverse events were most frequent in the 97.4 mg/m^2^ cohort (Table 4). Grade 3 or 4 laboratory hematologic toxicities were reported in 8 (18.6 %) patients across dosage cohorts. The most common grade 3 or 4 laboratory hematologic toxicity was lymphopenia, which occurred in 8 patients and across dosage cohorts (5.9, 29.6, 55.0, 97.4, and 126.6 mg/m^2^). Grade 4 leukopenia occurred in 1 patient in the 126.6 mg/m^2^ cohort. Grade 3 or 4 neutropenia also occurred in 2 patients in the 97.4 mg/m^2^ cohort (grade 3) and in 1 patient in the 126.6 mg/m^2^ cohort (grade 4).

**Table 4:** Grade 3/4 adverse events by cohort (no event occurring in >1 patient)^a^

3.0 mg/m^2^ *n* = 3	4.2 mg/m^2^ *n* = 3	5.9 mg/m^2^ *n* = 5	19.7 mg/m^2^ *n* = 3	29.6 g/m^2^ *n* = 4	55.0 mg/m^2^ *n* = 5	73.0 mg/m^2^ *n* = 3	97.4 mg/m^2^ *n* = 9	126.6 mg/m^2^ *n* = 2
• Abdominal pain • Arthralgia • Back pain	• Abdominal pain	• Hyperkalemia	• Back pain • Hypokalemia • Hyponatremia • Lumbar vertebral fracture	• Hemoglobin decrease • Hyponatremia • Lymphopenia • Pain in extremity • Pyrexia • Streptococcal sepsis	• Abdominal pain • Anxiety • Arthralgia • Back pain • Cellulitis • Constipation • Hypokalemia • Impaired healing • Musculo-skeletal pain • Peripheral edema • Scrotal edema • Somnolence	• Anemia • Leukopenia • Diarrhea • Decreased appetite • Musculo-skeletal pain • Neck pain • Headache • Pleural effusion	• Hemolytic anemia^b^ • Lymphopenia • Nausea • Vomiting • Pyrexia^b^ • Hyperbilirubinemia^b^ • Increased blood pressure • COPD	• Neutropenia^b^ • ECG change

^a^Cohorts with no adverse events reported (11.8 and 41.4 mg/m^2^) not shown

^b^Treatment-related

*COPD*, chronic obstructive pulmonary disease; *ECG*, electrocardiogram

Serious adverse events occurred in 12 patients; most were deemed unlikely or not related to CEP-11981. Three patients (1 patient in the 97.4 mg/m^2^ cohort and 2 patients in the 126.6 mg/m^2^ cohort) experienced serious adverse events that were categorized as possibly or definitely related to CEP-11981: pyrexia, hemolytic anemia, hyperbilirubinemia, dyspnea, neutropenia, ECG change, and chest discomfort. No deaths occurred during the study.

### Pharmacokinetics

**Fig. 2:**
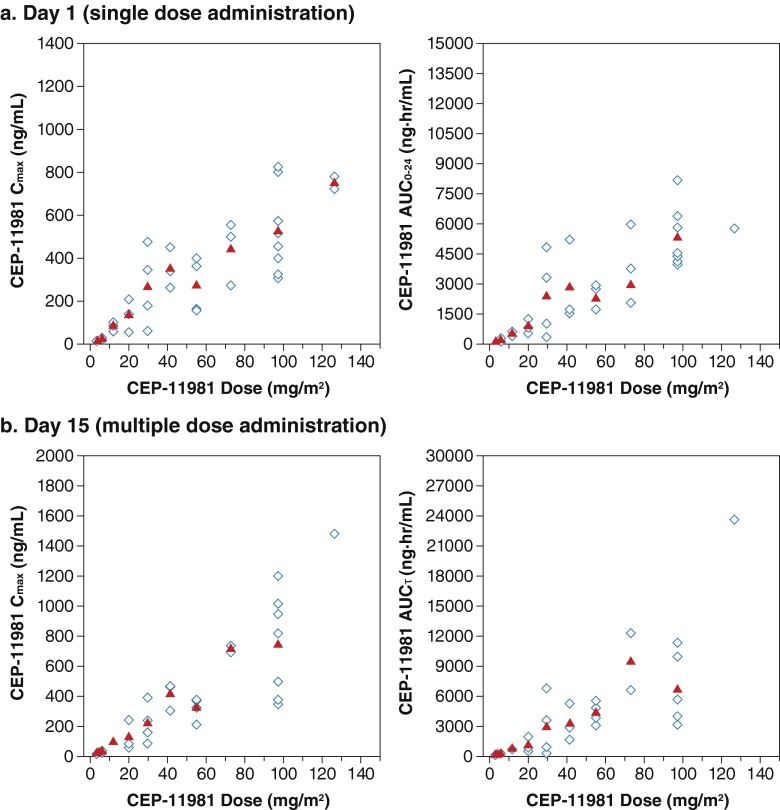
Individual Subject and Mean Values for C_max_ and AUC (*n* = 2–*n* = 9/dose level)^a^. Depicts that there is a relationship between drug dose and exposure (C_max_ and AUC) after administration of a single dose of CEP-11981 on day 1 (in Fig. 2a) and after multiple-dose administration of CEP-11981 at day 15 (in Fig. 2b). ^a^Open symbols represent the individual subject values; closed symbols represent the mean values. AUC_0–24_, area under the plasma drug concentration by time curve from 0 to 24 h; AUC_τ,_ area under the plasma drug concentration-time curve for 1 dosing interval; C_max_, maximum plasma drug concentration

**Fig. 3:**
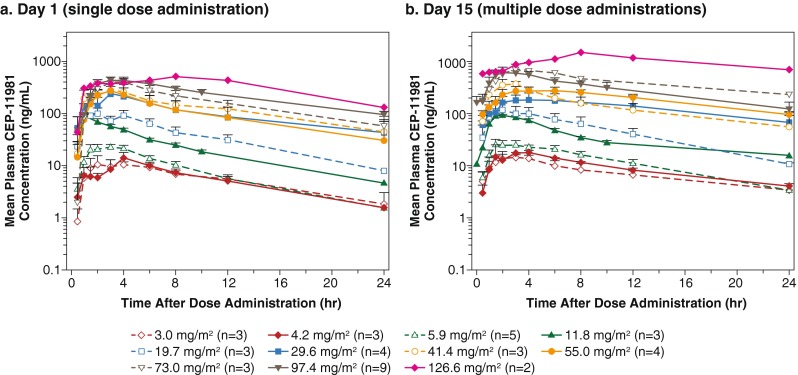
Mean (SE) Plasma Concentration-Versus-Time Profiles. Shows that some patients demonstrated a biphasic decline after achieving peak plasma concentration and other patients showed a monophasic decline after single-dose administration (Fig. 3a) and that the mean plasma concentration-versus-time profile after multiple-dose administrations of CEP-11981 (Fig. 3b) was qualitatively similar to that of single-dose administration. *SE*, standard error

There was a relationship between drug dose and exposure (C_max_ and AUC) after administration of a single dose (Fig. 2a). Dose proportionality could not be reliably assessed because of the extent of interpatient variability and small increments in dosages between the cohorts (Fig. 2a). There was interpatient variability of absorption after administration of a single dose, but there did not appear to be a relationship between dose and T_max_ after administration of a single dose across cohorts (Table 5). The mean plasma concentration-versus-time profiles after single-dose administration (Fig. 3a) showed that some patients had a biphasic decline after achieving peak plasma concentration, with an initial phase of drug distribution and a slower terminal elimination phase. Other patients had a monophasic decline, which was likely due to a prolonged period of absorption. The mean t_½_ after a single-dose administration was between 8 and 10 h (Table 5).

**Table 5:** Pharmacokinetic parameters following administration of CEP-11981 single dose and multiple doses by cohort (*N* = 43)^a^

	3.0 mg/m^2^ *n* = 3	4.2 mg/m^2^ *n* = 3	5.9 mg/m^2^ *n* = 5	11.8 mg/m^2^ *n* = 3	19.7 mg/m^2^ *n* = 3	29.6 mg/m^2^ *n* = 4	41.4 mg/m^2^ *n* = 3	55.0 mg/m^2^ *n* = 5^a^	73.0 mg/m^2^ *n* = 3	97.4 mg/m^2^ *n* = 9	126.6 mg/m^2^ *n* = 2
Single Dose											
Mean (SD) C_max_ (ng/mL)	13.7 (6.14)	16.8 (4.88)	26.0 (6.53)	84.9 (23.01)	135.7 (76.46)	266.9 (181.95)	351.9 (94.39)	251.9 (119.68)	444.2 (149.44)	526.4 (185.68)	751.2 (41.49)
Median (range) T_max_ (hr)	6.0 (2.0–6.1)	4.0 (1.0–6.0)	2.1 (1.5–4.0)	1.6 (1.5–3.0)	4.0 (1.0–4.3)	2.3 (1.0–3.1)	2.0 (1.0–4.0)	3.0 (3.0–4.0)	4.0 (3.0–4.1)	4.0 (2.0–6.0)	5.0 (2.0–8.0)
Mean (SD) AUC_0-24_ (ng•hr/mL)	127.0 (16.09)	119.3 (14.36)	194.8 (66.56)	516.7 (125.82)	874.0 (357.79)	2,376.3 (2,064.06)	2,820.3 (2,070.39)	2,274.0 (666.44)	3,938.7 (1,965.70)	5,320.4 (1,459.11)	5,767.0 (0)
Mean (SD) t_½_ (hr)	8.0 (2.82)	7.8 (3.69)	5.9 (1.06)	6.0 (3.66)	5.1 (1.33)	8.5 (5.48)	8.1 (4.54)	9.5 (2.57)	8.1 (2.19)	10.3 (4.59)	0 (0)
Mean (SD) R_pred_	1.2 (0.15)	1.2 (0.12)	1.1 (0.05)	1.1 (0.12)	1.0 (0.06)	1.2 (0.22)	1.2 (0.21)	1.2 (0.11)	1.2 (0.10)	1.3 (0.28)	0 (0)
Multiple Doses											
Mean (SD) C_max_ (ng/mL)	16.1 (2.35)	24.3 (9.23)	32.4 (8.40)	98.1 (5.64)	128.5 (98.34)	218.8 (130.35)	412.6 (93.94)	320.7 (75.09)	713.0 (30.38)	743.3 (336.75)	1,481.5 (0)
Median (range) T_max_ (hr)^b^	2.9 (1.5–4.0)	3.9 (1.5–4.0)	3.0 (1.3–6.0)	2.0 (1.6–2.1)	4.0 (1.5–4.0)	4.5 (2.0–8.0)	2.1 (1.7–3.1)	5.2 (3.0–8.0)	3.3 (3.1–3.5)	2.7 (1.5–6.0)	8.0 (8.0–8.0)
Mean (SD) AUC_τ_ (ng•hr/mL)	165.3 (82.98)	201.7 (46.72)	278.0 (69.45)	763.7 (128.14)	1,102.3 (734.79)	2,923.5 (2,939.79)	3,256.0 (1,816.44)	4,313.3 (1,091.33)	9,429.0 (3,993.74)	6,622.7 (3,282.62)	23,565.0 (0)
Mean (SD) t_½_ (hr)^c^	8.0 (2.92)	9.9 (4.55)	6.6 (1.51)	12.3 (9.70)	5.4 (1.20)	8.7 (5.74)	10.3 (6.33)	12.8 (4.32)	13.9 (9.48)	11.0 (6.24)	14.7 (0)
Mean (SD) R_obs_^d^	1.3 (0.62)	1.8 (0.64)	1.5 (0.59)	1.5 (0.15)	1.2 (0.29)	1.1 (1.24)	1.3 (0.46)	1.9 (0.19)	1.9 (0.14)	1.5 (0.85)	0 (0)

^a^1 patient was excluded from the multiple-dose pharmacokinetic analysis, *N* = 42 (*n* = 4 for the 55.0 mg/m^2^ dose)

^b^Overall median (range) for the multiple-dose pharmacokinetic analysis, *N* = 42: 3.0 (1.5–8.0)

^c^Overall mean (SD) for the multiple-dose pharmacokinetic analysis, *N* = 42: 9.9 (5.34)

^d^Overall mean (SD) for the multiple-dose pharmacokinetic analysis, *N* = 42: 1.5 (0.54)

*AUC*_*0*-*t*_, area under the plasma drug concentration by time curve from 0 h to last measurable drug concentration; *AUC*_*0*–*24*_, area under the plasma drug concentration by time curve from 0 to 24 h; AUC_τ,_ area under the plasma drug concentration-time curve for 1 dosing interval; *C*_*max*_, maximum plasma drug concentration; R_obs_, observed accumulation ratio; *R*_*pred*_, predicted accumulation ratio; *SD*, standard deviation; *t*_½_, terminal elimination half-life; *T*_*max*_, time to maximum plasma drug concentration

Pharmacokinetic parameters of CEP-11981 after the administration of several doses are summarized in Table 5, Figs. 2b and 3b. Multiple-dose administration with CEP-11981 also demonstrated a relationship between increased dosage and increased exposure (ie, C_max_ and AUC) (Table 5, Fig. 2b). Inter- and intrapatient variability after administration of multiple doses was also demonstrated, with no relationship between dosage and T_max_ after administration of multiple doses. The median T_max_ after multiple administrations ranged from 1.5 to 8 h across cohorts (Table 5). The mean plasma concentration-versus-time profiles after multiple administrations of CEP-11981 (Fig. 3b) were qualitatively similar to those after a single-dose administration (Fig. 3a). After multiple administrations, there were also patients who had a biphasic decline from the peak plasma concentration and other patients who had a monophasic decline; the mean t_½_ ranged from 8 to 10 h after multiple administrations of CEP-11981. The shapes of the mean plasma concentration-versus-time profiles for multiple- versus single-dose administrations showed that the absolute plasma concentrations were slightly higher after receipt of multiple doses reflecting the attainment of steady state.

### Pharmacodynamic bioassay

**Fig. 4:**
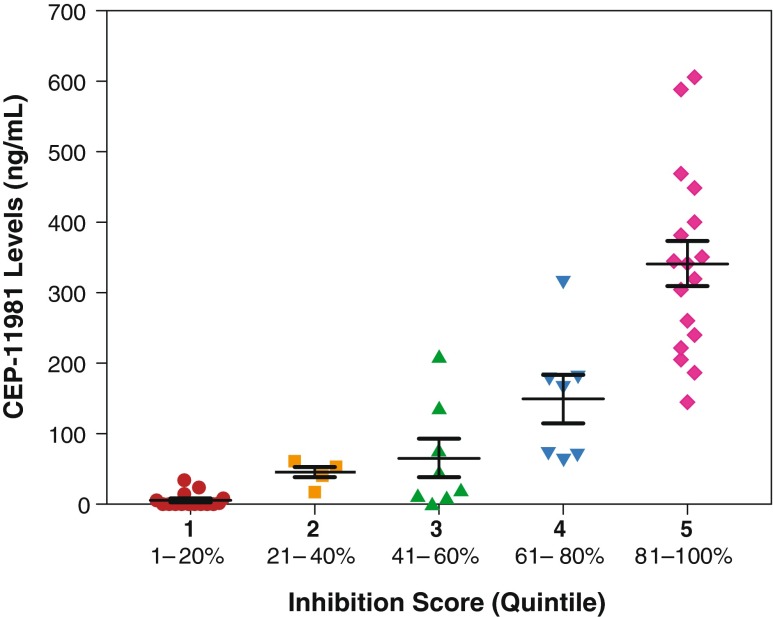
Ex Vivo Bioassay Analysis of Cellular VEGFR-2/KDR Kinase Inhibition in TrkA-VEGFR-2/KDR Chimeric PAE Cells (*N* = 27). Presents the magnitude of cellular VEGFR-2/KDR kinase inhibition using plasma samples from patients across all cohorts grouped by inhibition-score quintile. Normalized mean cellular VEGFR-2/KDR inhibition appeared to be exposure-related, although inhibition was variable. VEGFR-2/KDR, vascular endothelial growth factor receptor, subtype 2/KDR; PAE, porcine aortic endothelial

An ex vivo bioassay to evaluate the magnitude of cellular VEGFR-2/KDR kinase inhibition was conducted using plasma samples from the 27 patients across all cohorts who had samples with sufficient volume for analysis. The data were normalized relative to predose baseline levels of cellular VEGFR-2/KDR kinase inhibition. Less than 50 % inhibition of VEGFR-2/KDR kinase was achieved when using plasma samples from the lower dose cohorts (ie, 3.0, 4.2, 5.9, 11.8, and 19.7 mg/m^2^), which are shown in quintiles 1 and 2 of Fig. 4. Mean cellular VEGFR-2/KDR inhibition was observed beginning with the 29.6 and 55 mg/m^2^ dose cohorts (41 % to 60 % inhibition [quintile 3] and 61 % to 80 % inhibition [quintile 4]), respectively. Normalized mean cellular VEGFR-2/KDR inhibition was more pronounced (81 % or greater [ie, quintile 5]) and appeared to be exposure-related in plasma samples primarily in the 73.0, 97.4, and 126.6 mg/m^2^ cohorts, although inhibition was variable.

### Tumor response

**Fig. 5:**
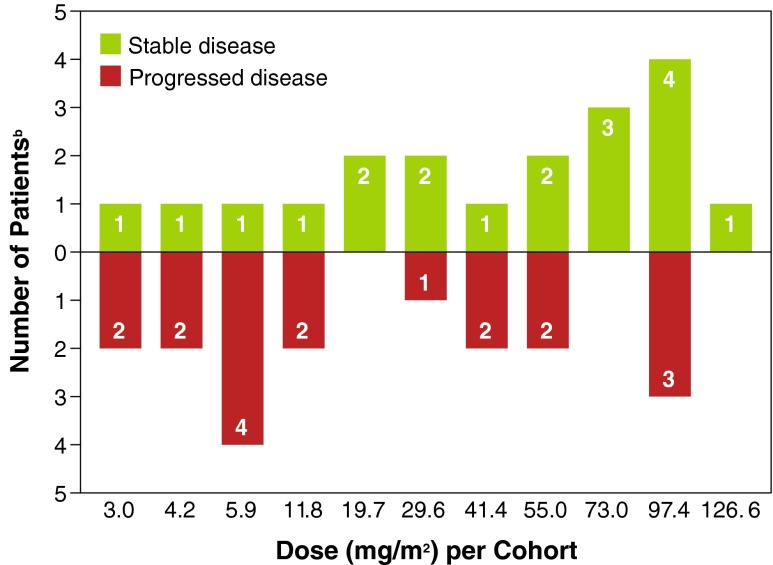
Best Overall Tumor Response Per Cohort^a^ at ≥6 Weeks. Depicts tumor response, with bars above the x-axis indicating patients (*n* = 19) who achieved stable disease and bars below the x-axis indicating patients (*n* = 18) with disease progression. ^a^No patient achieved complete or partial response. ^b^Six patients were not evaluable at dosages of 19.7, 29.6, 55.0, and 126.6 mg/m^2^ (*n* = 1, each); 97.4 mg/m^2^ (*n* = 2)

Out of 43 patients who received ≥ 1 dose of study drug, 37 patients were evaluated for tumor response. Although no enrolled patients had complete or partial response according to RECIST criteria, 19 of 37 (51 %) patients evaluated for tumor response had stable disease at ≥ 6 weeks; 18 had disease progression. The frequency of stable disease (defined as < 30 % decrease and < 20 % increase in the sum of the longest diameter of the target lesions) was higher in cohorts receiving doses ≥ 73.0 mg/m^2^ (8 of 14 [57.1 %] patients) compared with cohorts receiving ≤ 55.0 mg/m^2^ (11 of 29 [37.9 %] patients) (Fig. 5).

## Discussion

CEP-11981 was acceptably tolerated up to a dose of 97.4 mg/m^2^, which was determined to be the MTD. The appropriateness of this dose is consistent with findings ex vivo VEGFR-2/KDR inhibition in the quintile 5 plasma samples from patients receiving 97.4 mg/m^2^ (3 of 5 patients) and 126.6 mg/m^2^ (1 of 1) and that the best response (stable disease) occurred more frequently in cohorts receiving ≥ 73.0 mg/m^2^ (8 of 14, 57.1 %). In addition to the DLT of neutropenia in the patient treated at 126.6 mg/m^2^, 2 patients treated at 97.4 mg/m^2^ developed grade 3 neutropenia (1 patient in cycle 1 only, the other patient in a subsequent cycle only) that showed a temporal relationship to cycles of CEP-11981. Although the DLTs may be due to multiple targets of CEP-11981 (such as Aurora A), the importance of this observation is difficult to assess since only 3 of 9 patients treated at 97.4 mg/m^2^ received 2 or more cycles of treatment. Therefore, 97.4 mg/m^2^ was the MTD and can be considered a provisionally recommended phase II dose, but long-term tolerability remains to be determined.

Like CEP-11981, a number of other VEGFR-TKIs exhibited moderate pharmacokinetic variability in phase I and/or dose-escalation studies [27–30]. CEP-11981 potently and specifically inhibits VEGFR-1 and VEGFR-2, with IC_50_ values of 3 and 4 nM, respectively, in vitro [25]. In a previous study, targeted therapies were compared based on reported IC_50_ values for VEGFR-1 and VEGFR-2; sunitinib had IC_50_ of 10 nM (using murine NIH-3T3 cells) and 40 nM (using human umbilical vein endothelial cells [HUVEC]) for VEGFR-2 [31] and pazopanib had IC_50_ for VEGFR-2 of 8 nM (using HUVEC), while assays of VEGFR-1 were not done [32]. Pharmacodynamic bioassays showed inhibition of VEGFR-2 at doses with acceptable tolerability in this study. However, the neutropenia observed in patients at doses above 73.0 mg/m^2^, and its temporal relationship to CEP-11981 administration, suggest the inhibition of an unknown kinase. Laboratory studies have shown CEP-11981 inhibits Aurora A [25] and it is possible that this may account for the myelosuppression observed. There was intra- and interpatient variability after administration of multiple doses of CEP-11981 in patients with advanced solid tumors in this phase I study. The likelihood of a patient achieving responsiveness to therapy could not be predicted.

The recent controversy surrounding ponatinib highlights a growing body of evidence that most if not all TKIs sufficiently studied may increase patients’ risk of adverse cardiovascular events, including hypertension, thrombotic events, and QT-interval prolongation [33–35]. Nevertheless, product information for approved TKIs includes warnings about adverse cardiovascular events, and surveillance to determine how to best manage these risks is ongoing [33]. Some TKIs also are associated with treatment-related hepatotoxicity (as indicated by elevated ALT, AST, and total bilirubin) [36]. Labeling for lapatinib, pazopanib, and sunitinib, among others, includes warnings about hepatotoxicity [36]. In the present CEP-11981 study, 8 treatment-related events were reported. The most common grade 3/4 events affecting ≥ 5 % of patients across all groups were lymphopenia (19 %), neutropenia (7 %), and abdominal pain or back pain (7 % each). Regarding TKI-associated cardiovascular adverse events (QT interval prolongation, left ventricular dysfunction, and hypertension [34]), this CEP-11981 study reported 1 (2 %) patient with hypertension and 1 patient (2 %) with ECG changes. One patient receiving CEP-11981 (97.4 mg/m^2^) reported hyperbilirubinemia, which resolved after the patient discontinued the study. Overall, the tolerability profile of CEP-11981 appears to be promising; however, current evidence of efficacy is modest, and the compound is no longer in development by the sponsor.

In conclusion, CEP-11981 was well tolerated at doses between 3.0 and 97.4 mg/m^2^, with occasional routine dose reductions and with DLTs observed at 126.6 mg/m^2^. The MTD of CEP-11981 was determined to be 97.4 mg/m^2^. These events as well as DLTs and potentially treatment-related serious adverse events were generally reported in the highest-dose cohorts (97.4 [MTD] and 126.6 mg/m^2^). No patient experienced complete or partial response, 19 patients (44 % of the intent-to-treat population, or 51 % of patients with efficacy data) had stable disease at ≥ 6 weeks, primarily in the higher-dose cohorts. This study supports the dose-dependent biological effects of TKIs and the need for dose-finding efforts in order to achieve biological and clinical effective doses with this class of agents. The challenge of defining the optimal patient population for agents targeting the tumor microenvironment remains an opportunity.
